# High-Dose Barium Impaction Therapy Is Useful for the Initial Hemostasis and for Preventing the Recurrence of Colonic Diverticular Bleeding Unresponsive to Endoscopic Clipping

**DOI:** 10.1155/2013/365954

**Published:** 2013-05-09

**Authors:** Ryota Niikura, Naoyoshi Nagata, Kazuyoshi Yamano, Takuro Shimbo, Naomi Uemura

**Affiliations:** ^1^Department of Gastroenterology and Hepatology, National Center for Global Health and Medicine, 1-21-1 Toyama, Shinjuku, Tokyo 162-8655, Japan; ^2^Department of Radiology, National Center for Global Health and Medicine, 1-21-1 Toyama, Shinjuku, Tokyo 162-8655, Japan; ^3^Department of Clinical Research and Informatics, National Center for Global Health and Medicine, 1-21-1 Toyama, Shinjuku, Tokyo 162-8655, Japan; ^4^Department of Gastroenterology and Hepatology, Kohnodai Hospital, National Center for Global Health and Medicine, Chiba 272-8516, Japan

## Abstract

Most cases of colonic diverticular bleeding stop spontaneously, but some patients experience massive bleeding that requires emergency treatment. Endoscopy can be useful when the bleeding source is identified. However, bleeding sometimes recurs within a short period despite the successful endoscopic treatment. Under such conditions, more invasive therapy such as interventional angiography or surgery is required and can prolong hospitalization and involve frequent blood transfusions. We report the case of a 68-year-old woman who presented with massive hematochezia. The patient was in hemorrhagic shock and required 16 units of blood transfusion to recover to general condition. We performed multidetector row computed tomography, but it showed no sites of bleeding. We conducted colonoscopy and identified the source of bleeding as colonic diverticula. We treated the bleeding with endoscopic hemoclips and achieved hemostasis, but bleeding recurred the next day. Four units of blood transfusion were required. We tried high-dose barium impaction therapy to avoid further blood transfusion and surgery. No complications or recurrent bleeding was observed for an 18-month period. Therapeutic barium enema is an option for colonic diverticular bleeding unresponsive to endoscopic clipping and may be effective for preventing recurrent bleeding.

## 1. Introduction

Colonic diverticular bleeding is a common cause of lower gastrointestinal bleeding [1]. Although bleeding in approximately 80% of patients with colonic diverticular bleeding stops spontaneously [2], some patients continue to bleed or experience massive bleeding. Endoscopic treatment is effective when a stigma of recent hemorrhage (SRH) is identified [3]. However, endoscopic treatment has a high rate of recurrence within a short period in 38% of patients [4]. 

Barium impaction therapy is a noninvasive therapeutic modality that is useful when the active bleeding site has not been identified. Several reports have demonstrated favorable clinical outcomes of barium impaction therapy in patients with massive colonic diverticular bleeding [5–8].

We report a case of colonic diverticular bleeding successfully treated by barium impaction therapy which achieved initial hemostasis and prevented recurrent bleeding for at least 18 months following discharge. Barium impaction therapy was useful for improving clinical outcome and for avoiding further blood transfusion and surgery.

## 2. Case Report

A 68-year-old woman visited our emergency department with a complaint of large-volume, painless hematochezia over two days. The patient had type 2 diabetes, hyperlipidemia, and hypertension, and she had been treated for interstitial pneumonia. She also had a history of hematochezia 6 months earlier. She smoked 20 cigarettes/day for 49 years and had a history of 30 g/day of alcohol consumption. Hemoglobin concentration was 6.2 g/dL. The patient was in hemorrhagic shock and was transfused with 16 units of packed red blood cells. 

**Figure 1:**
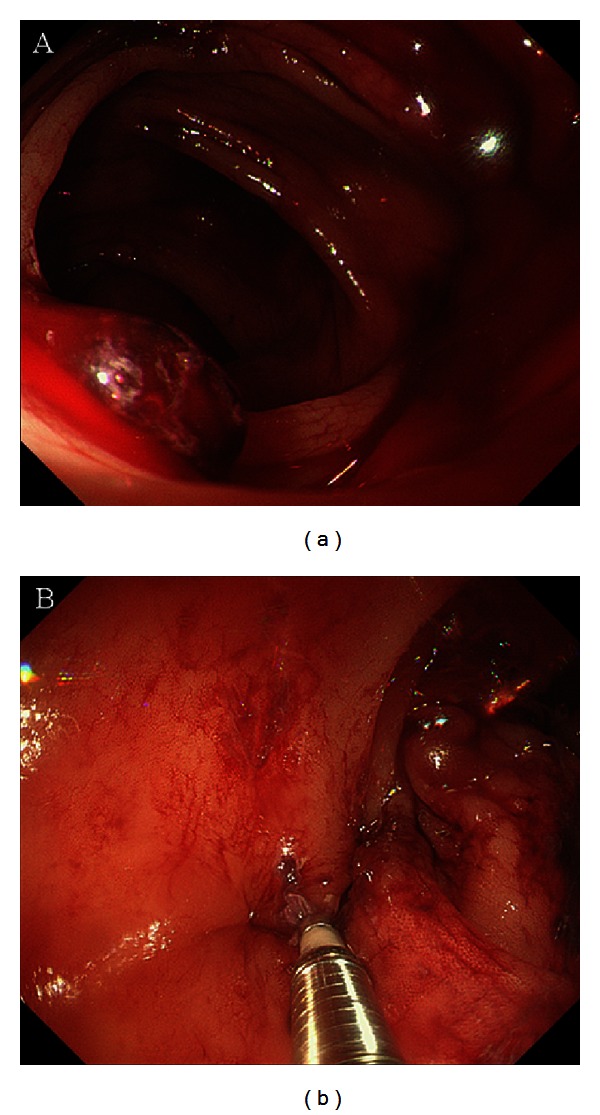
Colonic diverticular bleeding in the hepatic flexure on colonoscopy. (a) Stigmata of recent hemorrhage. (b) Endoscopic treatment using hemoclips.

**Figure 2:**
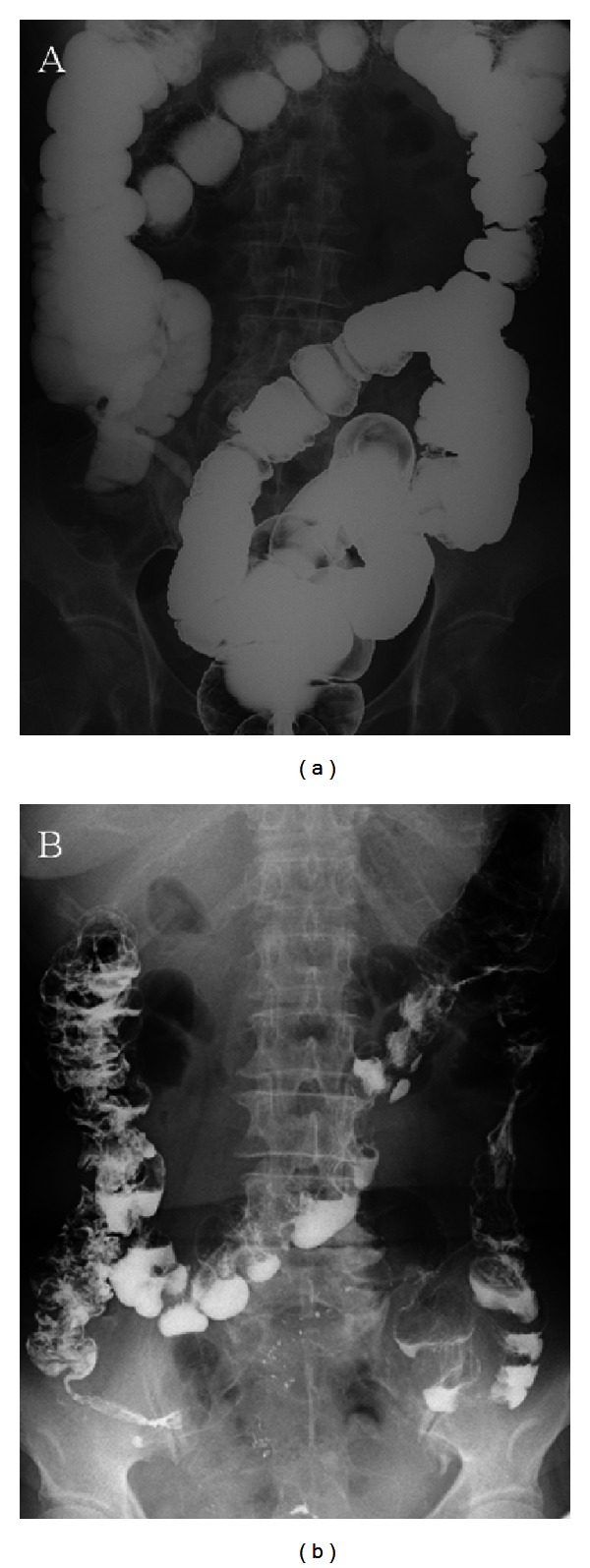
Barium radiography for colonic diverticular bleeding. (a) High-dose barium filling the entire colon by barium impaction therapy. (b) Abdominal radiograph showing barium retention in colonic diverticula taken the next day after barium impaction therapy.

Multidetector row computed tomography showed multiple diverticula in the ascending colon but no sites of bleeding. Colonoscopy was performed to detect the bleeding site and to reveal multiple diverticula in the ascending colon and blood clots in the diverticular dome. Colonic diverticular bleeding was diagnosed on the basis of blood clot detection in the diverticulum on colonoscopy (Figure 1(a)) [3]. Stigmata of recent hemorrhage (SRH) was treated using hemoclips (Figure 1(b)). Hemostasis was confirmed on colonoscopy, but rebleeding occurred the next day. A further 4 units of packed red blood cells were transfused. After obtaining informed consent, we performed transanal barium impaction therapy with 200% barium sulfate solution (400 mL) on day 7 (Figure 2). No complications occurred after the procedure, and hemostasis was achieved. The patient was discharged on day 13, and no rebleeding occurred for 18 months.

## 3. Discussion

Several studies on barium impaction therapy in colonic diverticular bleeding have been reported [5–8]. However, reports on the improvement of clinical outcome are limited [8]. Endoscopic treatment is effective when SRH is identified [3]. However, colonoscopy has shown a low detection rate for SRH and does not allow endoscopic hemostasis [9]. Barium impaction therapy on the other hand is useful when no SRH has been identified [6]. If neither endoscopic therapy nor barium impaction therapy can achieve hemostasis, then more invasive treatments such as interventional radiology (IVR) or surgery are required. However, such treatments may worsen the general condition and prolong both hospitalization and the frequency of blood transfusion [10, 11].

In the present case, colonic diverticular bleeding was diagnosed on the basis of SRH in the diverticular dome by colonoscopy [3]. However, endoscopic treatment could not achieve hemostasis. Jensen et al. [3] reported that in patients undergoing endoscopic treatment against SRH with epinephrine injection, bipolar coagulation had no recurrent bleeding. However, the rates of recurrent bleeding in other studies using endoscopic hemoclips and band ligation were as high as 11%–22% [12, 13]. It is currently controversial whether the endoscopic treatment is effective for preventing recurrent bleeding. Because of unsuccessful hemostasis by endoscopic treatment, we performed barium impaction therapy to avoid invasive therapy in the present patient. IVR and surgery are effective for arresting bleeding when endoscopic treatment fails [14]. However, IVR carries complications such as mesenteric infarction and myocardial infarction [10], while surgical intervention has a high mortality of approximately 33% [11]. We felt that IVR and surgery would be a high-risk option for the present patient because she had interstitial pneumonia and diabetes.

Although the mechanism of the hemostatic effect remains unclear, two factors can be considered: (1) the pressure induced by the barium solution tamponade of the bleeding vessel and (2) a direct hemostatic action due to the barium sulfate. Barium impaction in colonic diverticula often persists for weeks and sometimes months [15]. Barium administration may also influence the long-term hemostatic effect. Further studies are required to clarify the effectiveness and indication of barium impaction therapy in colonic diverticular bleeding.

In conclusion, the application of barium impaction therapy was effective for improving clinical outcomes such as achieving hemostasis and avoiding surgery and additional blood transfusion. Barium impaction therapy appears to be a viable option for colonic diverticular bleeding which is unresponsive to endoscopic therapy, and this modality may be an effective method for preventing recurrent bleeding.

## Abbreviations

IVR: Interventional radiology
SRH: Stigmata recent of hemorrhage.
